# Potentiation of Epidermal Growth Factor-Mediated Oncogenic Transformation by Sialidase NEU3 Leading to Src Activation

**DOI:** 10.1371/journal.pone.0120578

**Published:** 2015-03-24

**Authors:** Koji Yamamoto, Kohta Takahashi, Kazuhiro Shiozaki, Kazunori Yamaguchi, Setsuko Moriya, Masahiro Hosono, Hiroshi Shima, Taeko Miyagi

**Affiliations:** 1 Departments of Cancar Glycosylation Research, Institute of Molecular Biomembrane and Glycobiology, Tohoku Pharmaceutical University, Sendai, Japan; 2 Faculty of Fisheries and The United Graduate School of Agricultural Science, Kagoshima University, Kagoshima, Japan; 3 Molecular and Cellular Oncology, Miyagi Cancer Center Research Institute, Natori, Japan; 4 Departments of Cell Recognition Study, Institute of Molecular Biomembrane and Glycobiology, Tohoku Pharmaceutical University, Sendai, Japan; 5 Division of Cancer Molecular Biology, Graduate School of Medicine, Tohoku University, Sendai, Japan; Hungarian Academy of Sciences, HUNGARY

## Abstract

We previously demonstrated that sialidase NEU3, a key glycosidase for ganglioside degradation, is up-regulated in various human cancers, leading to increased cell invasion, motility and survival of cancer cells possibly through activation of EGF signaling. Its up-regulation is also important for promotion of the stage of colorectal carcinogenesis *in vivo* in human NEU3 transgenic mice treated with azoxymethane for the induction of aberrant crypt foci in the colon mucosa, accompanied by enhanced phosphorylation of EGF receptor (EGFR). To address whether the activation of EGF signaling by the sialidase is associated with oncogenic transformation, we here analyzed the effects of overexpression of NEU3 and EGFR in NIH-3T3 cells. When NEU3 was stably transfected with or without EGFR, it was associated with significant increases in clonogenic growth, clonogenicity on soft agar and *in vivo* tumor growth in nude mice either with or without the receptor overexpression in the presence of EGF, compared with the levels in their vector controls. Despite the fact that the endogenous level of EGFR is known to be extremely low in these cells, NEU3 significantly enhanced the phosphorylation of Akt and ERK, as well as that of the receptor. The NEU3-mediated activation was largely abrogated by the EGFR inhibitor AG1478 or PD153035, but significant clonogenic growth still remained. NEU3 was then found to activate Src kinase, and the clonogenicity was completely suppressed by an Src inhibitor, PP2. The activity-null mutants failed to activate Src and EGFR, indicating that ganglioside modulation by NEU3 may be necessary for the activation. NEU3 and Src were co-immunoprecipitated with EGFR in NEU3- and EGFR- transfected cells. These findings identify NEU3 as an essential participant in tumorigenesis through the EGFR/Src signaling pathway and a potential target for inhibiting EGFR-mediated tumor progression.

## Introduction

Sialidases catalyze the removal of sialic acid residues from the terminal positions of the carbohydrate groups of glycoproteins and glycolipids, which is the initial step in the degradation of these glycoconjugates. The sialidase reaction, therefore, is considered to influence many biological processes through alteration of the conformation and recognition of the biological sites of functional molecules. During malignant transformation, aberrant glycosylation has been observed as a characteristic feature of cancer cells [1, 2], and altered sialylation in particular is closely associated with metastatic potential and invasiveness. To shed light on the causes and consequences of such aberrant sialylation, our studies have focused on mammalian sialidases, which regulate the cellular sialic acid contents and function of glycoconjugates by desialylation [3].

Among four mammalian sialidases identified to date, the plasma membrane- associated and ganglioside-specific sialidase NEU3 appears to play particular roles in controlling transmembrane signaling by the modulation of gangliosides [4], and its aberrant expression is closely related to the pathogenesis of cancer [5]. We previously demonstrated that NEU3 is up-regulated in tumor compared with that in adjacent non-tumor tissues in colon, renal, prostate and ovarian cancers [6–9], which may be regulated by Sp1/Sp3 transcriptional factors [10]. NEU3 enhances cancer cell survival [6,11], cell migration and attachment [12], whereby it stimulates Ras activation with a consequent influence on ERK1/2 and Akt and actually enhances the EGF-stimulated tyrosine-phosphorylation of EGFR [11]. *NEU3* transgenic mice have also provided evidence of the involvement of this sialidase in carcinogenesis, in terms of an increase in azoxymethane- induced aberrant crypt foci [13], whereas *Neu3*- knock out mice exhibited reduction of tumor incidence in a colitis-associated colon carcinogenesis model induced by azoxymethane and dextran sodium sulfate [14]. These observations suggest the possibility that up-regulation of NEU3 is involved in not only augmentation of the malignant phenotype of cancer cells, but also in the process of malignant transformation.

EGFR is often found overexpressed or mutated in a wide variety of human cancers, and these events are closely linked to enhanced tumorigenicity [15]. The non-receptor tyrosine kinase, c-Src is also overexpressed in many of these same cancers, but is non-oncogenic in normal cells [16], implying that the two tyrosine kinases may functionally interact. In this context, it is assumed that co-overexpression of EGFR, c-Src and NEU3 in cancers is not just coincident but probably an essential event in tumorigenesis. To address this issue, we prepared stable transfectants of NIH-3T3 cells overexpressing NEU3 and/or EGFR, and examined them for their growth and tumorigenic properties. Here, we found that NEU3 potentiates EGFR-mediated tumorigenesis through the stimulation of EGFR phosphorylation and Src activity, and these findings should provide a new tool for the development of cancer therapy by the suppression of NEU3 that activates EGFR-signaling.

## Materials and Methods

### Cell culture and transfection

NIH-3T3 mouse fibroblast cells were obtained from Riken BRC Cell Bank (Tsukuba, Japan). The cells were cultured in Dulbecco’s modified Eagle’s medium (DMEM) supplemented with 10% fetal bovine serum (Invitrogen) at 37°C in a 5% CO_2_ atmosphere. For NEU3 overexpression, the entire open reading frame of the human *NEU3* gene [17] was inserted into the EcoRI site of a retrovirus vector pMXs-puro and the plasmid was introduced into PlatA. The target cells were then incubated with the culture media containing infectious viruses for two days and selected by cultivation in the presence of 2 μg/ml puromycin for 10–14 days, as previously described [18]. Null-activity mutants of NEU3, N88D and Y370C, were prepared as described previously [19] and subcloned into the EcoRI site of pMXs-puro. For EGFR overexpression, the human wild- type gene, which was kindly provided by Dr. M. Shibuya (Institute of Medical Science, University of Tokyo), was inserted into a retroviral vector pMXs-neo, and for selection, 800 μg/ml neomycin was used to obtain stable transfectants.

### Antibodies

Phospho-EGFR (Cell Signaling, Y-845, Y-1068), EGFR (Santa Cruz), phospho-ERK, ERK, phospho-Akt, Akt, phosphor-Src (Y416), Src, Fyn, Yes (Cell Signaling), and monoclonal anti-NEU3, prepared as described previously [20], were used in immunoprecipitation, or immunoblotting analysis.

### Quantitative RT-PCR analysis

Total RNA was prepared using an RNeasy mini kit (Qiagen) and reverse transcribed with SuperScript II (Invitrogen). Real-time PCR was performed with a QuantiTect SYBR Green PCR kit (Qiagen) and a Light Cycler PCR system (Roche). The primers for EGFR and NEU3 were as follows: mouse EGFR sense 5’-TTGGCCTATTCATGCGAA GAC-3’, and antisense 5’-GAGGTTCCACGAGCTCTCTCTCT-3’; human EGFR sense 5’-GACCTCCATGCCTTTGAGAA-3’, and antisense 5’-GCTGACGACT GCAAGAGAAA-3’; mouse NEU3 sense 5’-CTCAGTCAGAGATGAGGATGCT-3’, and antisense 5’- GTGAGACATAGTAGGCATAGGC-3’; and human NEU3 sense 5’-AGGTCAGTCTCCAGTACCTTC-3’ and antisense 5’-ACATCCAGCATCC TGACTGTAG-3’. The expression of glyceraldehyde- 3-phosphate dehydrogenase (GAPDH) was determined as an internal control.

### Sialidase activity assays

Crude extracts were used for sialidase assays using bovine brain ganglioside GM3 (Alexis Biochemicals, Lausen, Switzerland) as the substrate in the presence of 0.1%Triton X-100. After incubation at 37°C for 30 min, the amount of sialic acid released was determined by a modified thiobarbituric acid method or by fluorometric high-performance liquid chromatography with malononitrile [11]. One unit of activity was defined as the amount of enzyme that cleaved 1 nmol sialic acid from the substrates. Protein concentrations were determined by dye-binding assay (Bio-Rad Laboratories).

### Cell growth, colony formation and anchorage-independent growth assays

Cell growth rate was determined by 3-(4,5-dimethylthiazol-2-yl)-2,5- diphenyltetrazolium bromide assay using the WST-1 Cell Proliferation Assay System (Takara, Tokyo) on 96 well culture plates. For colony formation, cells were plated at 1000 cells/well in six-well dishes, cultured for 10–14 days and the colonies were counted using Gel-Doc (Bio-Rad) after staining with 0.1% crystal violet. Assays of colony formation in soft agar were performed as described previously [21]. Briefly, 1- mL underlayers consisting of 0.5% agar medium were prepared in six-well dishes by combining equal volumes of 1.0% Noble agar (Difco, Detroit, MI) with 2 x DMEM and 20% FBS. A total of 1 x10^5^ cells were suspended in 0.33% agar medium and then plated onto the previously prepared underlayers. After two to three 2–3 weeks, the colonies in five fields per sample were counted and the sizes per colony number were measured under a microscope.

### *In vivo* xenograft assays

Forty male athymic BALB/c nu/nu male mice (8–9 weeks old, SLC, Shizuoka, Japan) were used in this study. Animals were kept in rooms maintained at constant temperature (22±2 °C) and humidity (60%±15%) under a 12 h light- dark cycle, and were housed in ten groups (n = 3 or 4) in isolated ventilates cages and allowed free access to water and standard food. Mice were injected subcutaneously with the cells (1x10^7^) under ether anesthesia and then given either subcutaneous doses of murine EGF (Nacalai Tesque, 5 μg/day) every two days or no additional treatment. Tumor growth was measured every three days once tumors became visible. Tumor volume was estimated as follows: (length x width x thickness)/2. After 35–55 days of the cell injection, tumor-bearing mice were sacrificed under ether anesthesia. All animal experiments were performed in compliance with the Guidelines of Laboratory Animal Research, Tohoku Pharmaceutical University. The protocol was approved by the Animal Care and Use Committee of Tohoku Pharmaceutical University (Permit Number: A-13015-cn).

### Immunoprecipitation and immunoblotting

After culturing under serum-starved conditions for 16–24 h, cells were treated with EGF (20 ng/ml) for 15 min, washed with PBS and homogenized and solubilized by sonication for 10 sec in cold lysis buffer [50mM HEPES (pH7.5), 150 mM NaCl, 1% Nonidet P40, 2 mM EDTA, 7.5 μg/mL aprotinin, 10 μg/mL leupeptin, 10 mM NaF, 2 mM orthovanadate, and 2 mM PMSF]. After clarification by centrifugation (12,000 x g for 15 min), cellular lysates were immunoprecipitated with anti-EGFR antibody overnight, and then with protein A/G Sepharose beads (GE Healthcare Life Sciences) for 3 h. The immunocomplexes were then washed with cold lysis buffer, resuspended in SDS sample buffer, and subjected to SDS-PAGE and immunoblotting with the respective antibodies using ECL Plus Western blotting reagent (Amersham Biosciences). For the EGFR or Src inhibition, the cells were treated with specific inhibitor, AG1478 or PP2 (Calbiochem), respectively.

### Thin-layer chromatography

Glycolipids were extracted from cells as described elsewhere [7], fractionated by thin-layer chromatography on high-performance thin-layer chromatography plates (Baker, Phillipsburg, NJ, USA, or Merck, Darmstadt, Germany) and visualized with orcinol-H_2_SO_4_.

### Statistical analysis

The results are expressed as mean ± SD. All values were compared using Student’s t test.

## Results

### NEU3 promotes cell growth and colony formation of NIH-3T3 cells

**Fig 1:**
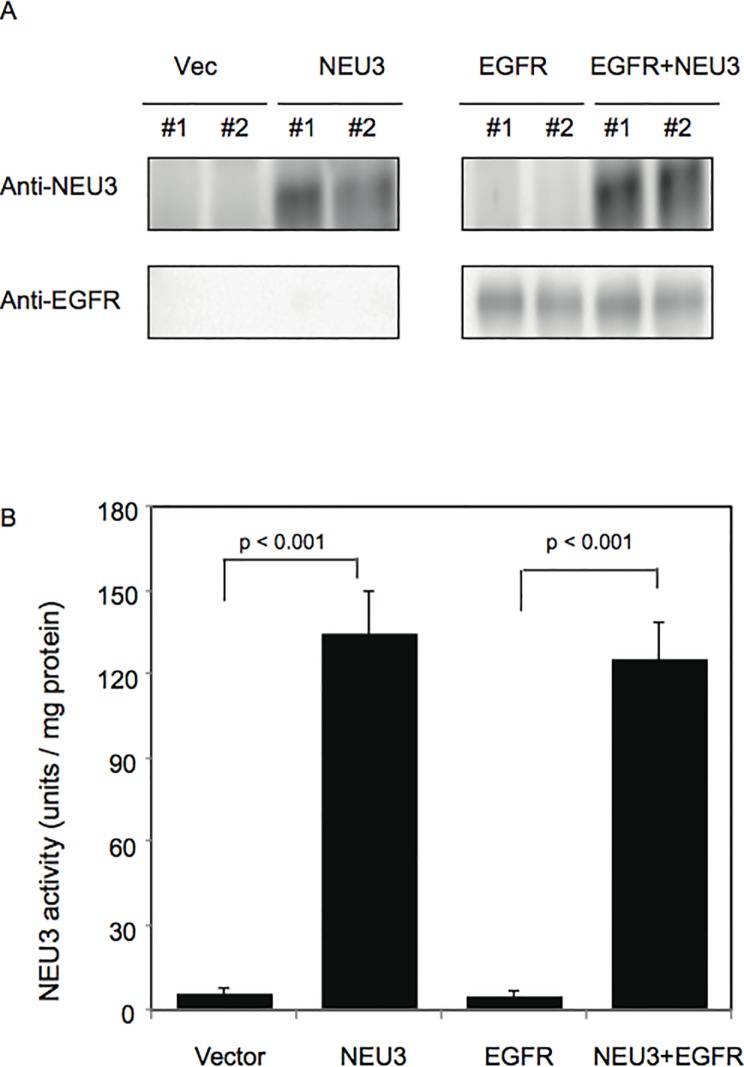
Measurement of NEU3 and EGFR levels in NEU3- and/or EGFR- transfected NIH-3T3 cells. (A) NEU3 and EGFR protein levels were analyzed by western blotting using antibodies for NEU3 and EGFR, respectively. The proteins were hardly detectable in the cells without the transfection of NEU3 and EGFR genes. Relative mRNA levels for murine EGFR/GAPDH were 0.7–0.9 in the controls and all the clones generated, and those for human EGFR/GAPDH were 0.1–0.2 in controls and NEU3-cells and 5,000–6,000 in the EGFR- and EGFR/NEU3-cells. (B) The sialidase activity levels toward mixed gangliosides (Bovine brain, Sigma) as a substrate were estimated. Only a slight sialidase activity was shown in vector controls and in EGFR-transfected cells.

To examine the effects of NEU3 on the cell proliferation and transformation, NIH-3T3 cells were utilized, since the cells were often employed in transfection assays for transforming genes [22]. We generated stable transfectants of the cells by the introduction of human NEU3 and/or EGFR cDNA using a retroviral vector system, and analyzed the clones (Vec-, NEU3-, EGFR-, and EGFR/NEU3-cells) for NEU3 and EGFR by western blotting with anti-NEU3 and anti-EGFR antibodies, respectively. A high level of NEU3 protein was detected in the NEU3-, and EGFR/NEU3-cells, but not in the vector controls or in the EGFR- cells (Fig. 1A), which is consistent with the data showing an approximately 25-fold increase in activity levels towards ganglioside substrates by NEU3 transfection compared with those in the controls (Fig. 1B). Such a potent increase of NEU3 expression is not unusual, but often seen in cancer compared with that in adjacent normal tissues, as we reported in the case of colon cancer [6]. The EGFR- and EGFR/NEU3- cells showed significant EGFR protein expression, whereas the controls and NEU3-cells did not, because of extremely low level of the endogenous EGFR in 3T3-NIH cells [23], confirmed by assessment of EGFR mRNA (Fig. 1A legend). The level of EGFR protein was hardly affected by NEU3 overexpression.

**Fig 2:**
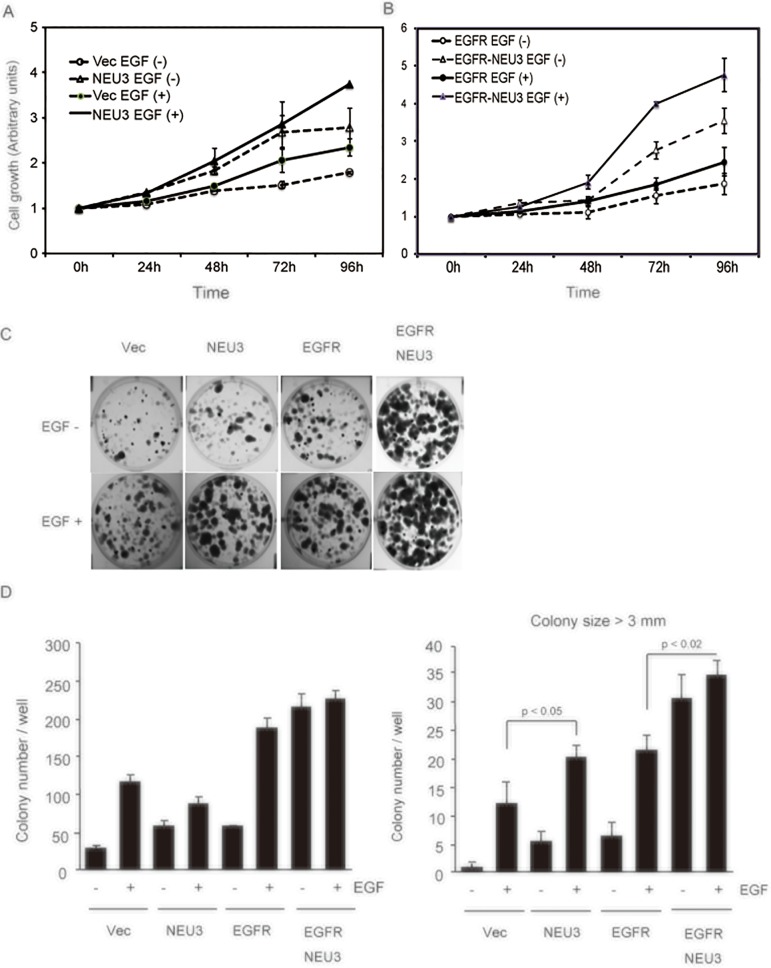
NEU3-mediated potentiation of cell growth assessed by MTT assays and colony formation assays. (A) The cell growth curves of NEU3-transfected cells were compared with those of vector controls in the absence and presence of murine EGF (20 ng/ml). Three independent experiments were performed (mean ±SD). (B) The cell growth curves of EGFR- and EGFR/NEU3-transfected cells are shown with or without EGF in independent experiments performed in triplicate (mean ±SD). (C) Colony formation assays in the transfectants. The cells were plated at 1000 cells/well in six-well dishes with or without EGF, and the colonies were quantified after 7–14 days of culture. Representative images are shown. (D) Values represent means with standard deviations obtained from three independent experiments. In the right graph, the number of colonies over 3.0 mm in size was counted in independent experiments performed in triplicate (mean ±SD).

We first determined the cell growth in NEU3-cells by MTT assays. NEU3 showed a growth rate higher than that of the vector controls, which was further enhanced in the presence of EGF (Fig. 2A); similar effects were also observed in the EGFR-cells (Fig. 2B). These clones were then examined in colony formation assays. As expected from the results of the MTT assays, NEU3 caused a significant increase in colony formation compared with that in the controls, even without EGF, which was markedly potentiated in combination with EGFR. Much greater enhancement of the above effects was observed upon EGF addition in all of these cells (Fig. 2C). The total colony numbers shown in Fig. 2D indicate that NEU3 and EGFR increased colony formation in monolayer culture and the overexpression of both had synergistic effects. Interestingly, the number of large colonies (over 3 mm in size) was increased by NEU3 to the same extent as by EGFR overexpression (Fig. 2D, right panel). These results indicate that NEU3 promotes colony formation in terms of size as well as number.

### NEU3 promotes anchorage-independent growth and *in vivo* tumorigenicity

**Fig 3:**
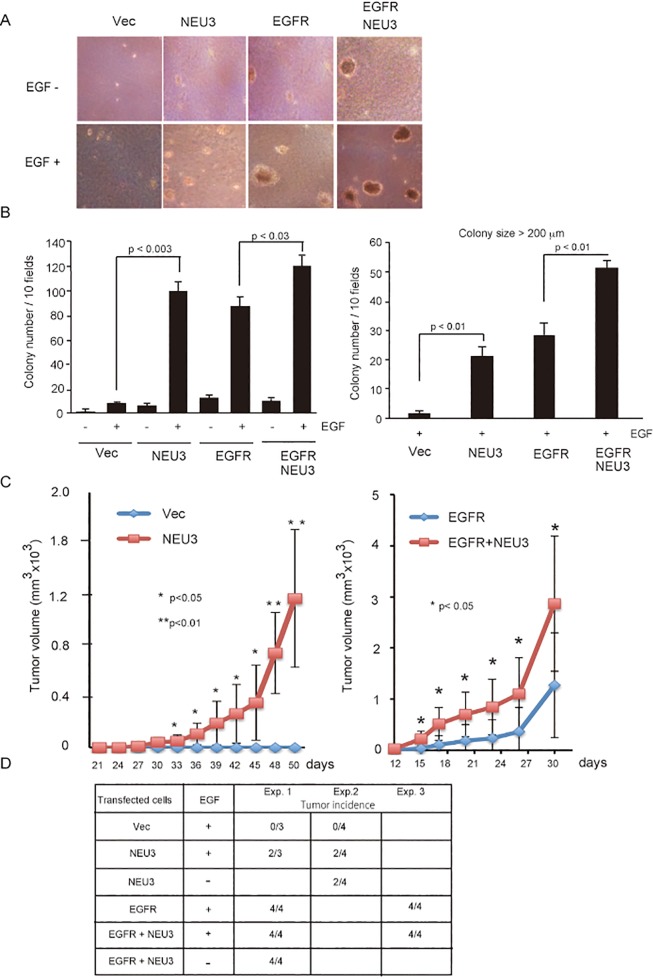
NEU3-mediated potentiation of anchorage- independent growth and in *vivo* growth in nude mice. (A) Anchorage-independent growth in soft agar. The cells (1 x 10^5^) were plated on soft agar with or without EGF, and two to three weeks later, the colony number and size were measured. Representative images are shown. (B) In the graph, the colony number on soft agar was calculated. The values are means with standard deviations obtained from three independent experiments. The graph in the right indicates the number of colonies over 200 μm in size. (C) *In vivo* growth in nude mice. Respective cells were subcutaneously transplanted into nude mice with injection of murine EGF (5 μg/ml) every two days, and the measured tumor weights are indicated. The results of experiments with the vector controls and NEU3-transfected cells (left graph) and those with EGFR- and EGFR/NEU3-transfected cells (right graph) are shown in the four groups (n = 3 or 4). (D) Tumor incidence in the mice transplanted with the cells. To confirm the results, other six groups (n = 4) of these cells were further examined with or without injection of EGF, as indicated in the lower table.

To determine the effects of NEU3 overexpression on the tumorigenic ability of the respective clones, we examined anchorage-independent growth. The NEU3-, EGFR- and NEU3/EGFR- cells apparently formed colonies in soft agar in the presence of EGF, but vector controls failed to do so (Fig. 3A). As observed in the monolayer culture, NEU3 potentiated the colony formation in soft agar to a considerable extent, similar to the case of EGFR, and led to a further increase when in combination with EGFR, although it had only a small effect in the absence of EGF (Fig. 3B). This is consistent with a previous report describing that EGF-induced growth in agar for human EGFR-transfected cells [23]. To determine further the effect of NEU3 expression on tumorigenesis *in vivo*, we injected the respective cells (1 x 10^7^ cells) subcutaneously into the flanks of male nude mice. The mice were given either subcutaneous doses of murine EGF (5μg) every two days or no additional treatment, although mature male mice have been shown to produce EGF [24]. As shown in Fig. 3C (left graph), 30 days after inoculation, interestingly, the NEU3-cells produced progressively growing tumors whereas the control cells did not form any visible tumors. In the mice injected with EGFR- and EGFR/NEU3- cells, the tumor growth was faster, the latency period was just less than half as long, and the average tumor volume was also greater than that of those received only the NEU3- cells (Fig. 3C, right graph). In particular, however, it is interesting that NEU3 markedly accelerated the tumor growth in volume and shortened the latency period in the mice injected with the EGFR/NEU3 cells compared with those with the EGFR-cells. As shown by the three experiments using independent clones (Fig. 3D), these results indicate that NEU3 potentiates the transformation of NIH-3T3 cells, and probably even possesses transformative ability alone, along with confirmation that EGFR is capable of transforming these cells. The transformation ability of NEU3- and/or EGFR- overexpressing cells was evident even in the case of no EGF injection, probably due to endogenous EGF in the male mice sufficient for the transformation. It would be also interesting to elucidate the cause of the results that NEU3- cells injected into some of the mice did not show *in vivo* tumorigenic potential.

### NEU3 activates EGFR signaling

**Fig 4:**
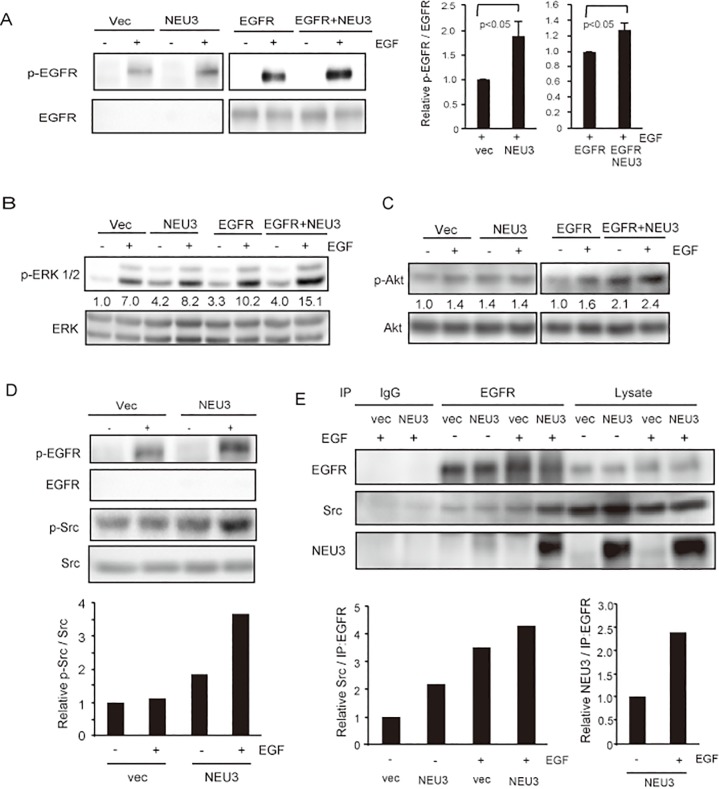
NEU3-mediated activation of EGFR/Src signaling. Phosphorylation of EGFR at Tyr-845 (A), ERK1/2 (B) and Akt (C) was determined with or without EGF by western blotting using respective antibodies. (A) Enhanced phosphorylation of EGFR at Tyr-845 in response to EGF and further synergistically by NEU3 overexpression. Results on immunoblotting are representative of three independent experiments, and the relative values for phospho-EGFR/EGFR in the presence of EGF were quantified in the right graph. (B, C) Enhanced phosphorylation of ERK1/2 (B) and Akt (C) with EGF and NEU3 overexpression. Each value shown under the blot represents as a value relative to that in the vector controls without EGF. (D) Enhanced Src activity by NEU3 overexpression. Results are representative of two independent experiments. The values for phospho-Src/Src relative to the vector controls without EGF are shown in the lower graph. (E) Immunoprecipitation of NEU3 and endogenous Src with anti-EGFR antibody and its promotion with EGF stimulation. Results are representative of two independent experiments. The values for the amounts of Src and NEU3, respectively, in the immunoprecipitates relative to those for the vector controls without EGF are shown in the graphs.

To understand how NEU3 affects EGF- dependent tumorigenesis and potentiates EGFR-mediated effects, we next examined EGFR signaling pathways in the respective cells. Consistent with our previous findings for HeLa cells [11], at 15min after EGF treatment, NEU3 increased EGFR phosphorylation even in the non-EGFR- transfected cells (Fig. 4A), presumably because of expression of a low number of analogous murine receptors in parent NIH-3T3 cells [23] and, besides, NEU3 transfection hardly changed the expression of the receptor (Fig. 1A legend). The effects of NEU3 were further potentiated by EGFR overexpression. The level of EGFR phosphorylation was higher at Tyr-845 than at Tyr-1068 in NEU3- transfected cells, as previously observed [11]. Stimulation of ERK and Akt phosphorylation was also observed by the forced NEU3 expression in the NEU3- and EGFR/NEU3- cells compared with that in the respective controls (Fig. 4B, C), although Akt phosphorylation was not notable in NEU3-cells compared to the controls. These cells were then examined to determine whether endogenous Src undergoes activation by NEU3, because the biological synergy between EGFR and c-Src has been reported [25]. The phosphorylation of Src was indeed increased by NEU3 overexpression as well as EGF addition (Fig. 4D) as quantified in the lower graph, suggesting the NEU3-dependent activation of both EGFR and Src. To verify that the NEU3-mediated activation occurs through the interaction of these molecules, we performed immunoprecipitation studies using anti-EGFR antibodies. NEU3 was found in the immunoprecipitates together with endogenous Src, and interestingly, EGF stimulation yielded a higher level of immunoprecipitable NEU3 and Src. These results suggest that NEU3 facilitates the activation of EGFR signaling by forming a complex with EGFR and Src (Fig. 4E). It should be noted here that endogenous c-Src in the complex with EGFR and NEU3 might be Src but not Fyn or Yes, ubiquitously expressed among the Src family kinases [26], based on our immunoprecipitation studies using antibodies specific to the respective proteins (data not shown).

### Inhibitors for EGFR and Src suppress colony formation

**Fig 5:**
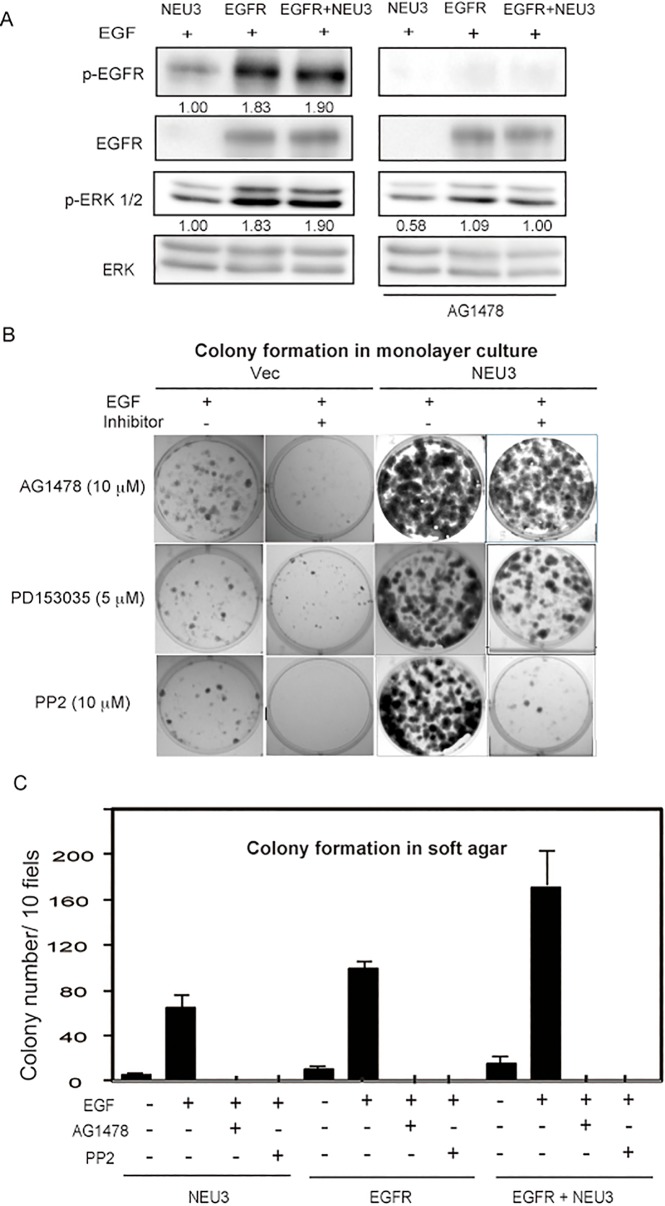
Suppression of colony formation and anchorage-independent growth by inhibitors. (A) Phosphorylation of EGFR and ERK1/2 was suppressed by an EGFR inhibitor, AG1478. Each value shown under the blot represents as a value relative to that in the vector controls. (B) Colony formation assays were performed in the presence of AG1478 (15 μM), PD153035 (5 μM) or an Src inhibitor, PP2 (10 μM). Representative images are shown. (C) Colony formation in soft agar was tested in the presence of AG1478 or PP2. Values are means with standard deviations obtained from three independent experiments (in graph)

To study the effects of EGFR and Src on colony formation in NEU3- and/or EGFR- overexpressing NIH-3T3 cells, the cells were treated with EGFR inhibitor AG1478 or PD153035, or with Src inhibitor PP2. With AG1478 (15 μM), EGFR phosphorylation was abrogated even in the cells forced to express EGFR, although ERK phosphorylation still remained to a certain extent (Fig. 5A). Under these conditions, NEU3 reduced the number of colonies but a considerable number still remained (Fig. 5B). Another EGFR inhibitor, PD153035 (5 μM), also caused the complete inhibition of EGFR phosphorylation (data not shown), but NEU3 resulted in the continued formation of some colonies (Fig. 5B). On the other hand, PP2 (10 μM) treatment resulted in entire suppression of colony formation, suggesting the importance of Src activity for the clonogenic growth. However, colony formation in soft agar was completely blocked by either AG1478 (15 μM) or PP2 (10 μM) in all of the cells (Fig. 5C), indicating that the activation of EGFR and Src is essential for anchorage-dependent cell growth.

### Ganglioside modulation by NEU3 is required for activation of EGFR signaling

**Fig 6:**
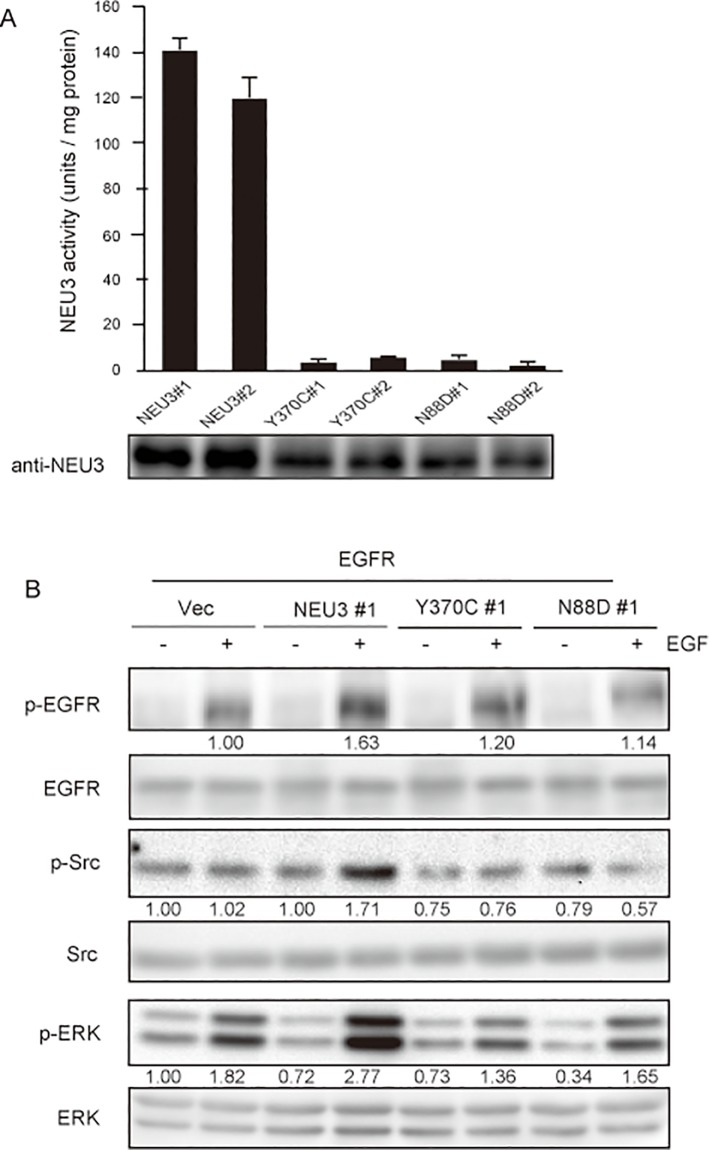
Requirement of NEU3 sialidase activity for enhanced phosphorylation of EGFR and Src. (A) The sialidase activities of NEU3 wild type and the mutant (Y370 or N88D) in the EGFR-overexpressing clones were assayed with ganglioside as a substrate in independent experiments performed in triplicate (mean ±SD). (B) The phosphorylation levels of EGFR, Src and ERK1/2 in the NEU3 wild and mutant clones in EGFR-cells were measured by western blotting using the respective antibodies. Each value shown under the blot represents as a value relative to that in the vector controls.

To investigate further the molecular mechanism behind the NEU3-mediated activation of EGFR and Src in the cell system, we generated NEU3 activity mutants and determined the phosphorylation of EGFR and Src in the wild-type NEU3/EGFR- or mutant NEU3/EGFR- cells. Mutations of the residues (N88D, Y370C) have been proposed to reduce the enzymatic activity markedly as the key catalytic residues [19, 27]. With ganglioside GM3 as a substrate, clones of the two mutants showed only a slight activity in the assays (Fig. 6A). In comparison of the phosphorylation levels among the cells, the wild-type NEU3 significantly increased the phosphorylation of EGFR together with that of ERK (Fig. 6B), whereas this failed in the mutants. It is particularly interesting that Src phosphorylation was enhanced only by the wild- type NEU3. These results indicate that NEU3-mediated activation of EGFR signaling requires NEU3 sialidase activity.

**Fig 7:**
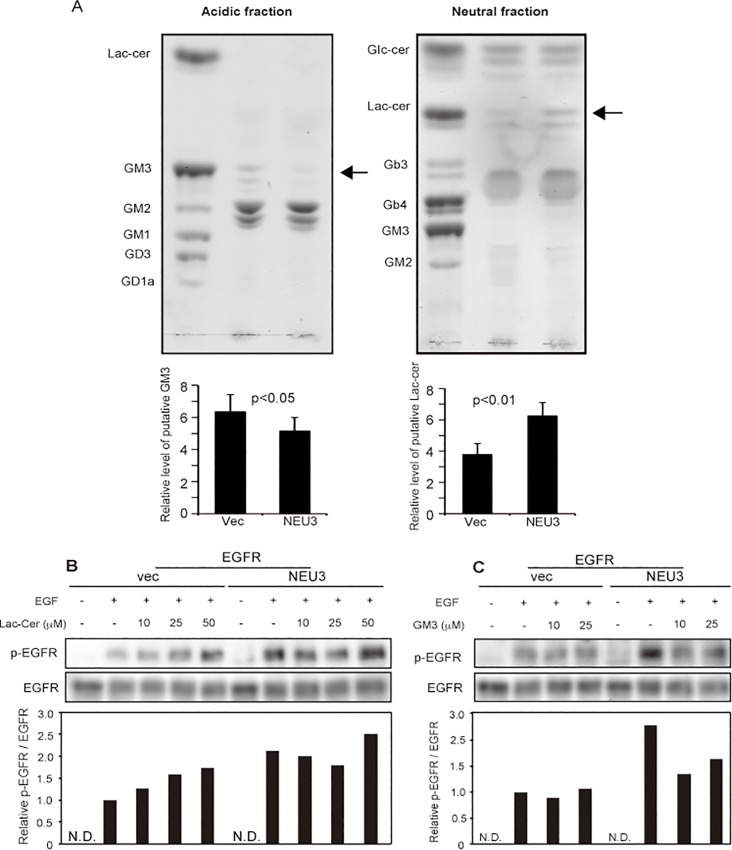
Alteration of glycolipids as a result of NEU3 catalytic reaction. Glycolipid changes due to NEU3 transfection were analyzed by thin- layer chromatography (A) in acidic (left panel) and neutral (right panel) fractions, and the relative changes in putative GM3 and Lac-cer were quantified in independent experiments performed in triplicate, as shown in the graphs below. (B, C) The effects of exogenously added Lac-cer (B) and GM3 (C) on EGFR phosphorylation in EGFR- and EGFR/NEU3-NIH-3T3 cells. Results are representative of two independent experiments. The values for phosphor-EGFR/EGFR relative to those for the vector controls with EGF are shown in the lower graphs.

As we previously demonstrated that human NEU3 is almost exclusively specific for gangliosides, alteration of the endogenous ganglioside pattern of NEU3-transfected NIH-3T3 cells was examined by thin layer chromatography (Fig. 7A). In the acidic fractions of the products, the level of the glycolipid with mobility similar to that of GM3 was reduced, whereas in the neutral fractions, the level of the glycolipid with mobility similar to that of lactosylceramide (Lac-cer) was increased. We have reported that Lac-cer stimulates EGFR phosphorylation [11], and other investigators have shown the evidence that GM3 reduces the phosphorylation [28–30]. To confirm the effects of the altered possible products, EGFR-transfected NIH-3T3 cells were treated with Lac-cer or GM3 under serum-depleted conditions. EGFR phosphorylation was increased by Lac-cer (Fig. 7B) and decreased by GM3 (Fig. 7C). All of the data together indicate that ganglioside- specific sialidase NEU3 potentiates EGF-mediated oncogenic transformation through EGFR and Src activation by ganglioside modulation.

## Discussion

The observation that the overexpression of both sialidase NEU3 and EGFR occurs in various human cancers suggested that they might functionally interact and contribute to cancer progression in a synergistic manner. Consistent with this idea, our previous results showed that NEU3 regulates the phosphorylation of EGFR and its dimerization in HeLa cells, leading to stimulation of the Ras cascade subsequent to the promotion of cell survival [11]. In the present study, using stable lines of NIH-3T3 cells that contain overexpressed wild- type human NEU3 and/or EGFR, we found that NEU3 is required for the biological synergy with the receptor as well as its phosphorylation, and additionally, for the activation of cellular Src, which has also been shown to be overexpressed in many of these same cancers. In the presence of EGF, NEU3 indeed accelerated cell growth, colony formation in soft agar and *in vivo* tumorigenicity in synergy with EGFR and Src through enhancing their phosphorylation. It has been reported that lung cancer- derived EGFR active- site mutants are constitutively active and thus oncogenic, even without EGF [31], and that this activation is probably caused by interaction with Src [32]. In this context, it is particularly interesting that the overexpression of NEU3 might give rise to cellular conditions similar to those for the mutant EGFR in terms of the promotion of anchorage independence and even *in vivo* tumorigenicity through enhancing the association with EGFR and Src, if EGF is present. The detection of NEU3 together with Src in the EGFR immunoprecipitates was more evident in the presence of EGF, indicating that the signal complex may facilitate the EGFR phosphorylation subsequent to the activation of downstream signaling. However, the detailed mechanisms behind the physical interaction of the three molecules remain to be elucidated.

The NEU3-mediated effects were dependent on the sialidase catalytic reaction, with the putative product Lac-cer possibly affecting the EGFR phosphorylation, as shown in the experiments with exogenously administered Lac-cer. On the other hand, in terms of the studies of EGFR regulation by GM3 to date, the evidence demonstrates GM3-mediated inhibition of EGFR phosphorylation by interaction of GM3 with N-glycan in the ectodomain of EGFR through carbohydrate- to -carbohydrate interactions [28–30], but there is a report showing the low binding ability of Lac-cer to the ectodomain [33]. We do not currently know whether glycolipids such as Lac-cer generated by the NEU3 reaction interact directly with EGFR. In contrast to the ectodomain inhibiting EGFR phosphorylation, the intracellular juxtamembrane region has been described as acting as an activation domain [34, 35]. Therefore, it is possible that Lac-cer interacts with this region, although the connection of Lac-cer, located in the external leaflet of the bilayer, with Src and the domain of EGFR, located in a cytoplasmic site of the membrane, is unclear. Alternatively, it is feasible that NEU3 could participate in the regulation of EGFR/Src signaling in the glycosphingolipid- enriched membrane microdomains, because of evidence for these three molecules being located in microdomains such as caveolae [20, 36].

In conclusion, the overexpression of NEU3 could cause the constitutive activation of EGFR together with Src activation in the presence of EGF, and subsequently potentiation of the tumorigenicity of cancer cells. This is in good agreement with our previous observations on NEU3 transgenic mice showing an involvement of NEU3 in colon carcinogenesis in terms of an increase in azoxymethane- induced aberrant crypt foci together with enhanced phosphorylation of EGFR in the colon mucosa [13], although some other such as Wnt-related molecules should also confer the tumorigenic potential in the case of colon cancer [37]. Furthermore, NEU3- dependent ERK activation would bring about NEU3 transcriptional activation, leading to positive feedback activation of EGFR signaling, because of ERK signaling elevating sp1 and sp3 transcription factor expression, which is responsible for NEU3 transcription [10]. To prevent the potentiation of tumorigenicity, suppression of the up-regulated NEU3 in cancer is particularly important. Thus, NEU3 may be a potential target for cancer therapy as a molecule upstream of the EGFR/Src pathway, since the therapeutic efficacies of inhibitors for EGFR and Src have not been so encouraging despite many clinical trials for current anticancer therapies.
